# Multi-resource collaborative optimization for adaptive virtual machine placement

**DOI:** 10.7717/peerj-cs.852

**Published:** 2022-01-06

**Authors:** Zhihua Li, Meini Pan, Lei Yu

**Affiliations:** 1 Department of Computer Science and Technology, Jiangnan University, Wuxi, Jiangsu, China; 2 IBM Research, Yorktown Heights, NY, USA

**Keywords:** Gaussian model, Collaborative optimization control, Resource utilization imbalance, VM placement

## Abstract

The unbalanced resource utilization of physical machines (PMs) in cloud data centers could cause resource wasting, workload imbalance and even negatively impact quality of service (QoS). To address this problem, this paper proposes a multi-resource collaborative optimization control (MCOC) mechanism for virtual machine (VM) migration. It uses Gaussian model to adaptively estimate the probability that the running PMs are in the multi-resource utilization balance status. Given the estimated probability of the multi-resource utilization balance state, we propose effective selection algorithms for live VM migration between the source hosts and destination hosts, including adaptive Gaussian model-based VMs placement (AGM-VMP) algorithm and VMs consolidation (AGM-VMC) method. Experimental results show that the AGM-VMC method can effectively achieve load balance and significantly improve resource utilization, reduce data center energy consumption while guaranteeing QoS.

## Introduction

Cloud data centers have stringent requirements for their operating environment, and the maintenance of the normal operation of a cloud data center usually results in high power consumption. Previous research (Li, Zhu & Zhao, 2017) indicates that all data centers consume more than 1.5% of the world’s electricity power in the world, and this percentage continues to rise, which leads to increasing operating costs and carbon emissions for cloud service providers. Physical machines (PMs) account for approximately 40% of the total energy consumption of a typical cloud data center, more than any other types of devices. Unfortunately, an IBM study pointed out that the average CPU utilization in cloud data centers is only 15% to 20% (Birke, Chen & Smirni, 2012). It is evident that low resource utilization contributes to unnecessary energy consumption in cloud data centers, and increasing resource utilization can reduce the number of running PMs and lower energy consumption. However, it has been reported (Voorsluys, Broberg & Venugopal, 2009) that high resource utilization often triggers an overloading risk of hosts, thereby degrading quality of service (QoS). Therefore, maintaining the resource utilization of PMs at a reasonable level can help balancing workload in cloud data centers, which in turn will reduce energy consumption while guaranteeing QoS.

In cloud data centers, live virtual machine (VM) migration technology allows VMs to be migrated from one host to another after a short downtime (Beloglazov, Abawajy & Buyya, 2012; Li, Yan & Yu, 2018), thereby balancing the load between different PMs. However, the process of VM migration, VM placement, and VM consolidation is subject to multiple resource constraints, in which multiple types of resources are correlated and mutually constrained. This makes it difficult to maintain a high utilization of all resource types simultaneously. This can potentially lead to resource utilization imbalance between different resources and overload risking, or even exhaustion of one or more resource types even while others are underused. Due to the excessive consumption of a single resource in hosts, the host overloading will emerge prematurely. This situation will trigger meaningless virtual machine migrations. It not only leads to the waste of a lot of resources, increases the energy consumption of the cloud data center, but impacts QoS. Hence, it should be avoided. Such resource utilization imbalance problem can readily spur wastage of the resources in a cloud data center. Maintaining a utilization balance between the different types of resources in a cloud data center not only helps keeping the utilization of those resources at a desirable level but also balances the workload of the cloud data center, and reduces energy consumption and unnecessary VM migrations.

In this paper, we propose a multi-resource collaborative optimization control (MCOC) approach to balance resource utilization. In our approach, PMs are first categorized into nine states according to the distribution of the utilization of multiple resources. A Gaussian model is used to estimate the probability that running PMs are in a balanced state of multi-resource utilization. Based on that, a set of algorithms are proposed for selecting the VMs to be migrated and their destination hosts, with the goals to minimize the migration time and maximize the probability of load balance state of the hosts. Finally, an adaptive Gaussian model-based VM consolidation (AGM-VMC) method that integrates these algorithms for VM consolidation is proposed. The experimental results of the proposed VM consolidation method on real data sets show that it effectively reduces the total number of VM migrations, decreases energy consumption and maintains the load balance of the cloud data center.

## Related works

VM migration has been exploited for load balancing and VMs consolidation, which aims to improve energy efficiency and quality of service (QoS) of data centers. VMs placement addresses the issue that where the migrated VMs come from and where to, thus is the key for VMs consolidation.

### VM placement based on heuristic packing

The VM placement problem (Younge, Von Laszewski & Wang, 2010; Verma, Ahuja & Neogi, 2008; Feller, Rilling & Morin, 2011) is usually formulated as a packing problem, in which the goal is to migrate VMs into a PM in such a way that the following two requirements are simultaneously satisfied to reduce energy consumption: (1) the total amount of resources requested by VMs deployed in the host does not exceed the total amount of resources allocated to the host; (2) the number of running PMs in the cloud data center is minimized. Most packing problems are solved approximately using heuristic algorithms (Li, Yan & Yu, 2018). For the VM placement problem, the work (Beloglazov, Abawajy & Buyya, 2012) improves the Best Fit Decreasing (BFD) algorithm and proposes an improved Modified Best Fit Decreasing (MBFD) algorithm to perform VM placement. First, the MBFD algorithm sorts the to-be-migrated VMs in the descending order of CPU resource utilization. Then, it sequentially seeks destination hosts for the VMs while guaranteeing minimum energy consumption of the destination hosts after VM placement. Another study (Farahnakian, Pahikkala & Liljeberg, 2015) improved the BFD algorithm and proposed a utilization prediction-aware BFD (UP-BFD) algorithm. It sorts VMs in the descending order of resource demands, and sequentially migrate VMs to the physical machine with the highest load; if the destination host becomes overloaded after VM placement or is predicted to be at a risk of overload in a future period, the physical machine with the next highest load is selected instead. By using the k-nearest neighbor method to predict the CPU utilization in a future period, the UP-BFD algorithm reduces unnecessary VM migration. In addition, the algorithm further reduces energy consumption by moving all the VMs deployed in the PMs with the least load and turning off these hosts. However, a heuristic algorithm based on the packing problem for VM placement mostly assumes that all PMs have identical hardware configurations. This is inconsistent with the typical situation of heterogeneous PMs in a cloud data center, thereby greatly compromising the practical effectiveness of the algorithm. A number of greedy algorithms (Feller, Rilling & Morin, 2011; Li, Yan & Yu, 2018; Beloglazov, Abawajy & Buyya, 2012; Farahnakian, Pahikkala & Liljeberg, 2015) have been used to solve the packing problem, but these algorithms are prone to be trapped in local optima earlier than expected.

### VM placement based on distribution workload model

Several works proposed VM placement approaches with exploiting patterns of resource load or statistics-like schemes. Mishra & Sahoo (2011) proposed a multi-resource vector computing based virtual machine placement (MV-VMP) algorithm. The operating steps of the MV-VMP algorithm are as follows: (1) it first classifies VMs and PMs into states of high, medium, and low resource utilization according to different resource thresholds; (2) it maps the current VMs and PMs into six different regions while preferentially placing the VMs into the physical machine in the same region; (3) if no suitable destination host is found in the same region, the search continues in the neighboring region until VM placement is completed. Another study (Shidik, Azhari & Mustofa, 2016) proposed a Gaussian Mixture Model Based Virtual Machine Placement (GMM-VMP) algorithm that operates as follows: (1) the historical workload measurements of the PMs are fitted, and the overload situations of the PMs are detected by a Gaussian mixture model based on the configured resources of the PMs; (2) whether the PMs are at risk of overload is determined according to the overload history, and then the physical machine at the lowest risk of overload is selected as the destination host for VM placement. Due to the high prediction accuracy of the Gaussian model, the GMM-VMP algorithm can reduce the frequency of VM migration in the cloud data center and decrease the overload risk of PMs. Melhem, Agarwal & Goel (2017) analyzed the resource utilization of PMs in a data center and proposed a Markov Prediction Model Based Power Aware Best Fit Decreasing (MPABFD) algorithm for VM placement. The MPABFD algorithm uses a first-order Markov chain to build a prediction model for resource utilization based on the resource distribution characteristics of the data center. Based on predicted resource utilization, the model classifies the future load state of each physical machine into three types: overload state, normal load state, and underload state. PMs that are predicted to be in a normal load state in the future are selected as the destination hosts for VM placement. Lastly, the destination hosts are sorted in the descending order of energy consumption, and the VMs are placed in the destination hosts with optimization of energy efficiency. In short, although VM placement based on statistics-like model can reduce the risk of host overload and reduce unnecessary VM migration, this method requires a large amount of historical load data to train the model, which increases the storage costs of a cloud data center.

### Intelligent algorithm-based VM placement

VM placement is subject to multiple resource constraints and requires an optimal balance of multiple goals. It has been pointed out (Li, Yan & Yu, 2018) that VM placement is an NP-hard problem. Since intelligent algorithms or intelligent evolutionary algorithms have certain advantages in solving such problems, a number of approaches based on that have been proposed for VM placement. A genetic algorithm (GA) and a First-Fit (FF) algorithm were combined to solve the VM placement problem (Hallawi, Mehnen & He, 2016) as follows: (1) first, to reduce energy consumption and improve resource utilization, an optimization model is established to minimize the number of running PMs and minimize the amount of unused resources; (2) second, the GA chromosomes are encoded with gene bits to denote PMs and the information on the gene bits to denote VMs, and each VM is assigned to a physical machine using the FF algorithm; (3) finally, the optimal mapping between VMs and PMs is obtained by continuously and iteratively updating the population and selecting the optimal chromosome sequence according to the optimization model. However, minimizing the number of running PMs and minimizing the resource surplus could be contradictory goals. Therefore, optimizing both objectives simultaneously can easily cause the algorithm to be trapped in local optimization. Some studies (Alharbi & Tian, 2019; Farahnakian, Pahikkala & Liljeberg, 2015; Ferdaus, Murshed & Calheiros, 2014) have employed ant colony algorithms to solve the VM placement optimization problem. These methods generate random-state transition rules by defining the local heuristic information of artificial ant colonies and updating the pheromones accumulated during the iterative process. These rules are used to select the destination host where the VMs to be migrated for VM placement. A Particle Swarm Optimization (PSO) algorithm is a bionic intelligent algorithm, and Pandey et al. (2010) proposed a Particle Swarm Optimization Based Virtual Machine Placement (PSO-VP) approach. This approach, which has the optimization objective of minimizing energy consumption of the data center, solves the optimization model using the PSO algorithm. Compared with the Best Resource Selection (BRS) algorithm, PSO-VMP reduces the energy consumption of the data center, significantly decreases the operating costs, and optimizes resource utilization. Feng, Wang & Zhang (2012) developed a resource allocation model based on a PSO algorithm and used the model in conjunction with Pareto theory to solve the problem of resource allocation in a data center during VM placement.

Compared with the heuristic and statistics-like methods, intelligent algorithms can usually obtain an approximate optimal solution, thus they are more effective in dealing with the complicated workload imbalance problem in VM placement. However, they are also greatly influenced by the optimization model. A suitable optimization model can help guide an algorithm’s evolution and improve its convergence speed. However, the algorithm needs to be iterated during the evolution process, which leads to long execution time and low efficiency.

## Adaptive estimation of the probability of dynamic workload

### Workload imbalance between multiple resources

**Figure 1:**
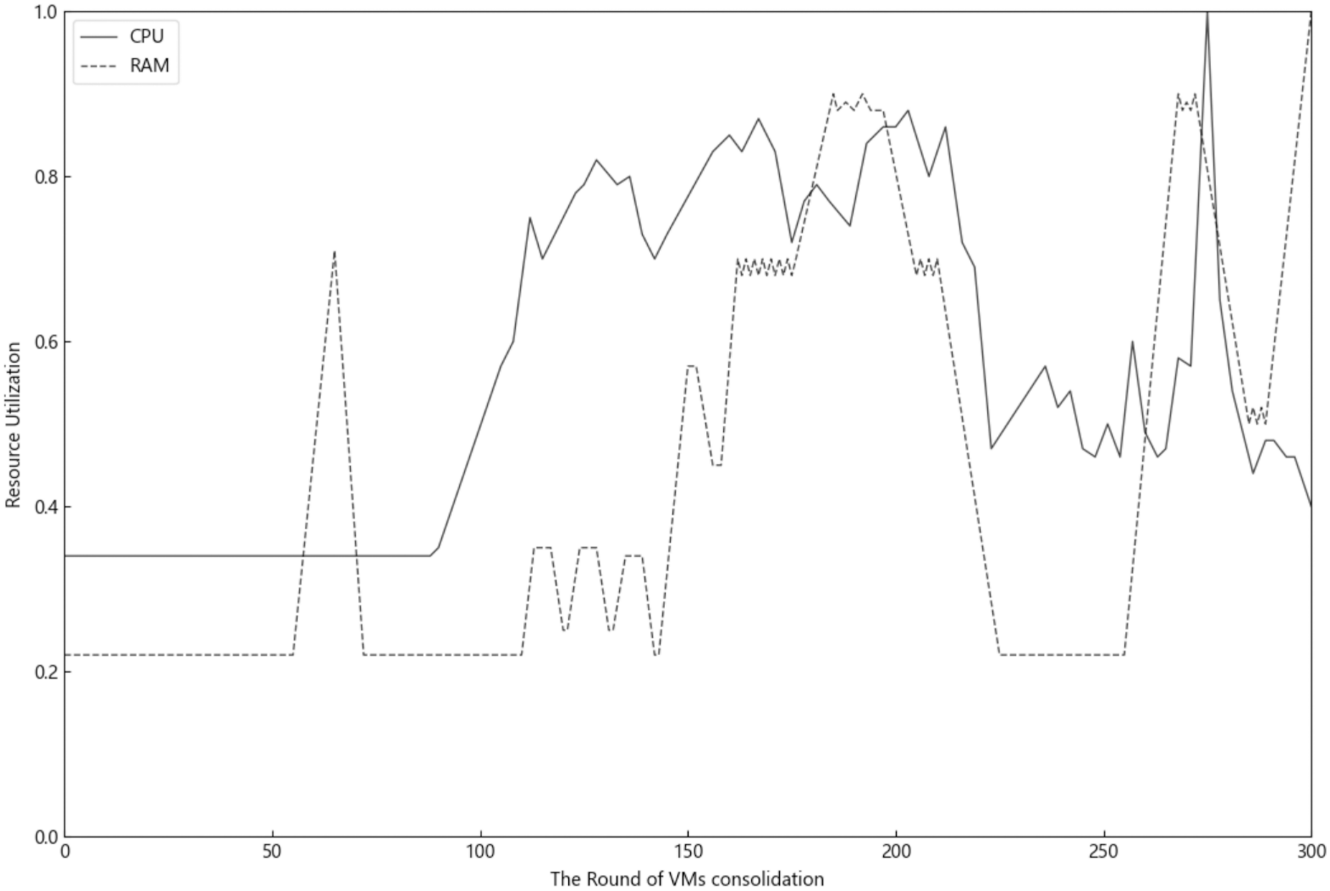
The inconsistency of multiple resource utilization of PMs.

Modern cloud data centers generally contain a variety of computing resources such as CPU and memory. Figure 1 shows the actual changes in the utilization of CPU and memory resources of a running physical machine in the data center over a 24-h period, of which the data of Fig. 1 come from Alibaba Cluster Data V2018 (https://github.com/alibaba/clusterdata/blob/master/cluster-trace-v2018/trace_2018.md). The data center collects resource utilization data from the physical machine every 5 min, for a total of 288 data points obtained over a 24-h period. As VM consolidation cycle changes, the utilization of one type of resources differs greatly from that of another type, even within the same period. Moreover, the utilization of different resources peaks at different times of the day. This observation demonstrates a real scenario in which wastage of physical resources occurs several times during a day while the objective of optimizing utilization of multiple types of resources is not met. Since VMs request multiple resources during VM consolidation, peak utilization of any resource in the physical machine would place the physical machine at a higher overload risking. Therefore, controlling the utilization of multiple resources within a desirable range would effectively reduce the resource wastage in the data center and mitigate host overloading risk.

PMs can be classified into three states based on their CPU utilization: low CPU load, normal CPU load, and high CPU load. An excessively low CPU load is wastage of the resource, while an excessively high CPU load will likely result in an overloading of PMs when CPU resource requests from VMs suddenly increase. This will then affect the QoS of the data center. The possibility of such undesirable situation can be minimized to the greatest extent only when the CPU load is maintained in a normal state prior to the request. Similarly, other data center resources such as memory can be classified into underload, normal-load, and high-load states. As suggested above, load balance can be achieved in the data center to reduce resource wastage only when the different resources are simultaneously in a normal state of utilization. This study proposes a VM consolidation mechanism and algorithm for coordinated optimization and control of multiple resources in physical machines. It aims to reduce resource wastage arising from unbalanced resource utilization in physical machines and maintain the utilization of multiple resources at a reasonable level.

### Gaussian model for the state of balanced resource utilization

**Figure 2:**
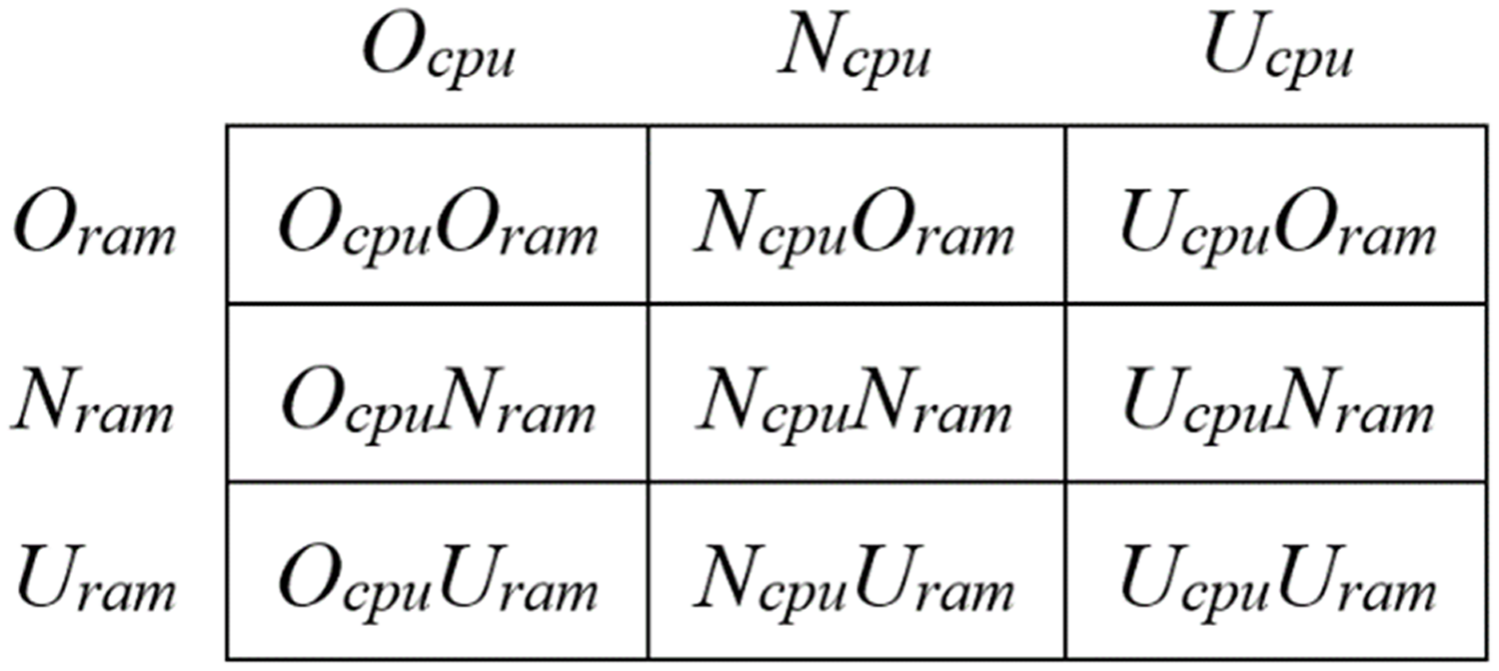
The resource state combination matrix of the PMs.

Suppose a running physical machine has two resources, CPU and memory, which are denoted by $R = \left\{ {cpu,ram} \right\}$. The running physical machine is considered to be in a state of overload ( ${O_r}$), a state of normal load ( ${N_r}$), or a state of underload ( ${U_r}$) depending on the degree of resource utilization, where $r \in R$. ${O_r}$ represents the state in which the utilization of resource $r$ in the physical machine exceeds the overload threshold. ${N_r}$ represents the state in which the utilization of resource $r$ is between the underload threshold and the overload threshold, namely the normal load state. ${U_r}$ represents the state in which the utilization of resource $r$ is lower than the underload threshold. A dual-threshold method is adopted here to determine the load state of the physical machine. Specifically, the overload threshold is denoted as ${T_{over}}$ and the underload threshold is denoted as ${T_{under}}$. When the resource utilization of the physical machine is greater than ${T_{over}}$, the physical machine is in an overload state; when the resource utilization is less than ${T_{under}}$, the physical machine is in an underload state; when the resource utilization is greater than ${T_{under}}$ but less than ${T_{over}}$, the physical machine is in a normal state. The utilization of a given resource is always in one of the three states, and thus, the joint utilization of two resources is always in one of the nine states (Fig. 2). It means that the resource utilization of two resources in the physical machine during the entire operational period switches between the nine states.

The load of a running physical machine is essentially the sum of all the resources allocated to VMs operating on the physical machine, with the load increasing or decreasing as the resources are requested or returned by the VMs. VM-requested resource utilization is derived from the task requests that are submitted by users to the data center, and these requests are random; this causes the resource utilization requests of VMs to be random. As analyzed in Section of “Workload imbalance between multiple resources”, when the total resource utilization of the physical machine is at the ${N_{cpu}}{N_{ram}}$ state, the physical machine is in a balanced workload state and reaches the optimal synergy of resource utilization, with the data center achieving the most reliable QoS. The ${N_{cpu}}{N_{ram}}$ state is referred to as the balance state of host resource utilization. Although it is difficult to predict accurately, studies (Yu, Chen & Cai, 2016; Zheng, Li & Dong, 2016; Sharma & Saini, 2016) have shown that the resource utilization of VMs follows the Gaussian distribution. Accordingly, the Gaussian distribution is adopted in this paper to estimate the probability of resource utilization of VMs, to further calculate the probability of PMs being in the ${N_{cpu}}{N_{ram}}$ state.

Let the resource utilization of $v{m_i}$ for resource $r$ be $X_{v{m_i}}^r$, which follows the Gaussian distribution, namely $X_{v{m_i}}^r: N({\mu _r},{\sigma _r}^2)$. ${\mu _r}$ and ${\sigma _r}$ are the expected value and standard deviation of the historical records of $X_{v{m_i}}^r$, respectively, which can be calculated according to Eq. (1) and Eq. (2) respectively.

(1) $${\mu _r} = \displaystyle{1 \over n}\sum\limits_{i = 1}^n {u_t^r}$$

(2) $${\sigma _r}^2 = \displaystyle{1 \over n}\sum\limits_{i = 1}^n {{{(u_t^r - {\mu _r})}^2}}$$

where, $n$ is the number of historical requests, $u_t^r$ is the ratio of the $v{m_i}$-requested amount of resource $r$ at time $t$ to the allocated amount of resource $r$ in the physical machine.

Further, the cumulative probability of $X_{v{m_i}}^r$is estimated according to Eq. (3),

(3) $$\varphi  _r^i(x|{\mu _r},{\sigma _r}) = \displaystyle{1 \over {{\sigma _r}\sqrt {2\pi } }}\int\limits_{ - \infty }^x {{e^{ - \textstyle{{{{\left( {x - {\mu _r}} \right)}^2}} \over {2{\sigma _r}^2}}}}dx}$$

where $0 \le x \le 1$, and $\varphi  _r^i(x)$ denotes the probability that at any moment, the ratio of the $v{m_i}$-requested amount of resource $r$ to the allocated amount of resource $r$ in the physical machine does not exceed $x$.

Consequently, the probability that the host ${h_j}$ at any moment is in the ${N_{cpu}}{N_{ram}}$ state, *i.e*., the probability that the resource utilization of the physical machine is in a balanced state, is $f({h_j})$. This can be calculated using Eq. (4),

(4) $$f({h_j}) = \sum\limits_{r \in R} {{\alpha _r} \times \left\{ {\displaystyle{1 \over m}\sum\limits_{i = 1}^m {\left[ {\varphi  _r^i\left( {{T_{over}}} \right) - \varphi  _r^i\left( {{T_{under}}} \right)} \right]} } \right\}}$$

where $m$ represents the total number of VMs deployed in ${h_j}$. In Eq. (4), ${T_{over}}$ is the overload threshold, and ${T_{under}}$ is the underload threshold, they are empirically configured. When the resource utilization is greater than ${T_{under}}$ but less than ${T_{over}}$, the physical machine is in a normal state. $\varphi  _r^i\left( {{T_{over}}} \right) - \varphi  _r^i\left( {{T_{under}}} \right)$ represents the probability that the host ${h_i}$ is in the normal load state. The probability that the VMs deployed in ${h_j}$ are in the ${N_{cpu}}{N_{ram}}$ state is first calculated using Eq. (3), and then the probability that host ${h_j}$ is in the ${N_{cpu}}{N_{ram}}$ state is estimated according to the resource utilization requests of the VMs. ${\alpha _r}$is the weight of resource $r$, which can be varied. The larger the value of ${\alpha _r}$, the greater the impact of the current load of resource $r$ on $f({h_j})$.

If CPU resource utilization of the physical machine is relatively high at a time when the memory resource utilization is low, the CPU resource will be more likely to be overloaded than the memory resource at that time. It suggests that the CPU resource has a larger impact on the host load than the memory resource. Accordingly, ${\alpha _{cpu}}$ should be greater than ${\alpha _{ram}}$, that is, ${\alpha _r}$ is proportional to the utilization of resource $r$ at the current moment. As analyzed in Section of “Workload imbalance between multiple resources”, the utilization of different resources of a physical machine during its operation is dynamic, and therefore ${\alpha _r}$ should be allowed to vary with the load during VM consolidation. Moreover, given the continuity of the load changes of the physical machine, ${\alpha _r}$ can be approximated using Eq. (5),

(5) $$\alpha _r^t = a \cdot \alpha _r^{t - 1} + (1 - a){\alpha _r}^\prime$$

where $\alpha _r^t$ represents the impact factor of resource $r$ in the current VM consolidation cycle, $\alpha _r^{t - 1}$ is the impact factor of resource $r$ of the previous VM consolidation cycle, ${\alpha _r}^\prime$ is the impact factor of resource $r$ calculated using the resource load status in the current VM consolidation cycle, and $a$ is a learning parameter, with $0 < a < 1$.

As shown in Eq. (5), the impact factor of resource $r$ in the current VM consolidation cycle is mostly decided by the load status of the current consolidation cycle when $a$ approaches zero. When $a$ approaches 1, it is mostly decided by the load status during the previous VM consolidation cycle. Here, $a$ is an empirical parameter, obtained by the experimental training results of the proposed algorithms.

To determine ${\alpha _r}^\prime$ in Eq. (5), and suppose the total impact of all resource loads in the physical machine on the host operation is 1, namely, $\sum\limits_{r \in R} {{\alpha _r}^\prime } = 1$. Further, to keep the optimal synergy of resource utilization of ${N_{cpu}}{N_{ram}}$, it should ensure the coordination and synchronization of different resource changes, identically satisfying ${{{u_{cpu}}} \mathord{\left/ {\vphantom {{{u_{cpu}}} {\alpha _{cpu}^{\prime}}}} \right.} {\alpha _{cpu}^{\prime}}} = {{{u_{ram}}} \mathord{\left/ {\vphantom {{{u_{ram}}} {\alpha _{ram}^{\prime}}}} \right.} {\alpha _{ram}^{\prime}}}$. Thereby the parameter ${\alpha _r}^\prime$ can be determined according to Eq. (6).

(6) $$\left\{ \matrix{{\alpha _{cpu}}^\prime = \displaystyle{{{u_{cpu}}} \over {{u_{cpu}} + {u_{ram}}}} \cr {\alpha _{ram}}^\prime = \displaystyle{{{u_{ram}}} \over {{u_{cpu}} + {u_{ram}}}}} \right.$$

where ${u_{cpu}}$ and ${u_{ram}}$ represent the CPU resource utilization and memory resource utilization of physical machine ${h_j}$ during the current VM consolidation cycle, respectively.

### Computing probability of normal state for hosts

The key issue to resolve first in the VM placement process is how to efficiently and quickly identify the source and destination hosts involved in a VM migration. Let $f\left( {{h_i}} \right)$ be the probability for host ${h_j}$ to be in the ${N_{cpu}}{N_{ram}}$ state. With the historical records of resource utilization of VMs, $f\left( {{h_i}} \right)$ can be computed by Eqs. (4)–(6). If $f\left( {{h_i}} \right)$ is higher during the next VM consolidation cycle after a virtual machine is migrated from it or a migrated VM is deployed to it, then host ${h_j}$ is selected as the preferential source host or the preferential destination host, respectively. Accordingly, $f({h_j})$ can be estimated as follows.

**Algorithm 1:** Determining probability of normal state

1: Input: *h*_*j*_,
2: Output: $f({h_j})$
3: $a$ = 0.2
4: If ${\alpha _{cpu}}$ and ${\alpha _{ram}}$ not existing
5: Initial ${\alpha _{cpu}} = 0.5$ and ${\alpha _{ram}} = 0.5$
6: End if
7: *VM*-list ← get the VMs in ${h_j}$
8: Caculate *each vm*^*’s*^ $\mu$ use Eq. (1) and $\sigma$ use Eq. (2) with *Virtual machine request history*
9: *Gaussian model ← each vm*^*’s*^ $\mu$, $\sigma$
10: Update ${\alpha _{cpu}}$ and ${\alpha _{ram}}$ use Eqs. (5) and (6) with the current resource utilization
11: Calculate $f({h_j})$ use Eqs. (3) and (4)

$f({h_j})$ can serve as a quantitative metric to guide the selection and placement of migration VMs, since $f\left( {{h_i}} \right)$ can be maximized by VM migration from host ${h_j}$, or deploying VMs to host ${h_j}$.

## Adaptive gaussian-based vm consolidation

### Overloading host detection

Overloading hosts are one of the main factors causing the QoS degradation of a data center. This section proposes a Gaussian model based multi-resource overloaded detection (GMMHOD) algorithm for detection of overloading hosts. The algorithm first develops a Gaussian model using Eqs. (1) and (2) based on the historical loads of multiple resources in PMs, and then uses Eq. (3) to estimate the overloading probability of PMs. Given the randomness of physical machine load, a physical machine is considered to be at a high overloading risk when the utilization of its resource $r$ exceeds 1 − ${\sigma _r}$, where ${\sigma _r}$ acts as reserved resource to accommodate the utilization fluctuations of resource $r$ in the physical machine. Using Eq. (3), one can compute $p_r^{over}$, the cumulative probability of the utilization of resource $r$ in the physical machine in the interval $[1 - {\sigma _r},1)$. The physical machine is considered to be at a high overloading risk when $p_r^{over}$ > 0.5. The pseudocode of the GMMHOD algorithm is described as follows.

**Algorithm 2:** GMMHOD

1: Input: *h*_*j*_
2: Output: *isoverload*_*i*_
3: For each *vm*_*i*_ in *VM*-list do
4: Calculate ${\mu _r}$ use Eq. (1) and ${\sigma _r}$ use Eq. (2)
5: *probability*_*r*_ *= probability*_*r*_ + ( $\varphi _r^i(1|{\mu _r},{\sigma _r}) - \varphi _r^i(1 - {\sigma _r}|{\mu _r},{\sigma _r})$)
6: End for
7: *probability*_*r*_ = $probabilit{y_r}/m$
8: If *probability*_*r*_ > 0.5 then $isoverloa{d_i} \leftarrow true$
9: else $isoverloa{d_i} \leftarrow false$
10: End if

### VM selection

When a physical machine in a data center is at high overloading risk, some VMs need to be migrated from the physical machine to avoid overload and consequent degradation of QoS. To maximize the probability that the host is in the ${N_{cpu}}{N_{ram}}$ state, it is necessary to select the VM whose outward migration will maximize $f({h_j})$ of host ${h_j}$. That is, outward migration of such a VM will make the host more likely to be in the ${N_{cpu}}{N_{ram}}$ state compared with other VMs. In the meantime, VM memory size should be considered when selecting a VM, as VM migration causes VM downtime, with a larger VM memory leading to a longer duration of suspended VM operation (Farahnakian, Ashraf & Pahikkala, 2015) and thereby a greater negative impact on QoS of data center. Therefore, when selecting a VM to migrate, in addition to maximizing the host’s $f({h_i})$, it is necessary to prefer the VM with smaller memory. The priority of VMs for migration is computed using Eq. (7) based on the current $f({h_i})$ of PMs and the memory size of VMs. On this basis, a Gaussian model based minimum migration time (GMMMT) algorithm is proposed as a VM selection algorithm for migration.

(7) $$S({h_j},v{m_i}) = {\raise0.7ex\hbox{${f({h_j}^{ - v{m_i}})}$} \!\mathord{\left/ {\vphantom {{f({h_j}^{ - v{m_i}})} {ra{m^{v{m_i}}}}}}\right.} \!\lower0.7ex\hbox{${ra{m^{v{m_i}}}}$}}$$

where $S({h_j},v{m_i})$ represents the migration priority of $v{m_i}$ on host ${h_j}$, $f({h_j}^{ - v{m_i}})$ the probability that ${h_j}$ is in the ${N_{cpu}}{N_{ram}}$ state when $v{m_i}$ is migrated from ${h_j}$, and $ra{m^{v{m_i}}}$ the memory size occupied by $v{m_i}$.

As suggested by Eq. (7), the smaller the memory size occupied by the VM and the higher the probability that the physical machine workload is in the ${N_{cpu}}{N_{ram}}$ state, the higher the migration priority of the VM. The pseudocode description of the GMMMT algorithm is as follows.

**Algorithm 3:** GMMMT

1: Input: ${h_j}$
2: Output: $vm$
3: *probability* = −1
4: $vm = null$
5: For each *vm*_*i*_ in *VM*-list do
6: Calculate $S({h_j},v{m_i})$ use Eq. (7)
7: If $S({h_j},v{m_i})$ > probability then
8: *vm* = *vm*_*i*_
9: *probability* = $S({h_j},v{m_i})$
10: End if
11: End for

### VM placement

Each VM to be migrated should be placed in a suitable destination host. The first requirement for a suitable destination host is that it has sufficient resources. In a multi-resource constrained VM placement problem, if a VM requests a large amount of CPU but a small amount of memory, it is easy to deploy the VM in a physical machine with a small amount of available CPU and a large amount of available memory resource is prone to overload the CPU resource. In contrast, placing the VM in a physical machine with a large amount of available CPU and a small amount of available memory can not only effectively avoid host overloading but also improve memory resource utilization of the physical machine. In addition, it is desirable to select the physical machine that will have the highest probability of ${N_{cpu}}{N_{ram}}$ state after inward migration of VMs to the destination host. Therefore, we propose to compute the priority of destination hosts to be selected, shown in Eq. (8), according to the amount of unallocated resources in each physical machine and the probability of each host being in a state of balanced resource utilization. Migrated VMs are deployed in destination hosts sorted in decreasing order of priority during each cycle of VM consolidation.

(8) $$P({h_j},v{m_i}) = {\raise0.7ex\hbox{${f({h_j}^{ + v{m_i}})}$} \!\mathord{\left/ {\vphantom {{f({h_j}^{ + v{m_i}})} {\sum\limits_{r \in R} {\displaystyle{{{q_r}} \over {{c_r}}}} }}}\right.} \!\lower0.7ex\hbox{${\sum\limits_{r \in R} {\displaystyle{{{q_r}} \over {{c_r}}}} }$}}$$

where $P({h_j},v{m_i})$ represents the priority of ${h_j}$ as a destination host for $v{m_i}$, and $f({h_j}^{ + v{m_i}})$ represents the probability that host ${h_j}$ is in the ${N_{cpu}}{N_{ram}}$ state after placing virtual machine $v{m_i}$ in ${h_j}$; $f({h_j}^{ + v{m_i}})$ is proportional to the VM placement priority, that is, the higher the probability that the physical machine is in a state of balanced resource utilization after the virtual machine is placed, the higher the placement priority of the physical machine; ${q^r}$ is the quantity of resource $r$ requested by the $v{m_i}$ to be migrated, and ${c_r}$ is the amount of resource $r$ unallocated in host ${h_j}$.

As suggested by Eq. (8), the smaller the ratio of VM-requested resources to unallocated resources in a destination host, the more available resources the destination host can provide for the VM, the less likely the destination host is to be overloaded after the VM is placed in it, and thus the higher placement priority assigned to the destination host. Given the above context, an adaptive Gaussian Model based minimum overload probability (GMMOP) algorithm is proposed as a destination host selection algorithm. Its pseudocode description is as follows.

**Algorithm 4:** GMMOP

1: Input: *vm*_*i*_, *host-list*
2: Output: *host*
3: *host = null*
4: *probability* = −1
5: For each *h*_*j*_ in *host-list*
6: If *vm*_*i*_ cannot be placed on *h*_*j*_ then continue
7: Calculate $P({h_j},v{m_i})$ use Eq. (8)
8: If $P({h_j},v{m_i})$ > probability then
9: host = *h*_*j*_
10: *probability* = $P({h_j},v{m_i})$
11: End if
12: End for

### VM consolidation

An adaptive Gaussian model based VM consolidation (AGM-VMC) scheme is further proposed integrating the GMMHOD, GMMMT, and GMMOP algorithms. The working steps of the AGM-VMC method are as follows: (1) first, the scheme computes the overloading probability of running PMs using the GMMHOD algorithm, and includes the PMs at high risk of overload in the collection of overloaded PMs; (2) secondly, the method uses the GMMMT algorithm to perform live VM migration one by one from the overloaded PMs until the overload risk is eliminated, and adds the migrated VMs to the collection of outward migrated VMs; (3) thirdly, the method uses the GMMOP algorithm to find suitable destination hosts for the outward migrated VMs, namely those which satisfy the resource needs of the outward migrated VMs, and the low-overload-risk needs of PMs after VM placement; (4) finally, the method uses a greedy algorithm to iteratively select PMs with the lowest load, moves all of their VMs to suitable destination hosts, and turns off or hibernates them until the VMs on any running physical machine cannot be fully moved to any other running physical machine. Additionally, when there are many candidate destination hosts, multiple virtual machines are migrated through “one by one” scheme continuously to one or multiple different destination hosts during each cycle of VM consolidation.

The pseudo code of AGM-VMC method is described as below.

**Algorithm 5:** AGM-VMC

1: Input: *host-list*
2: Output: Map <*host, vm*>
3: For each *h*_*j*_ in *host-list*
4: If GMMHOD (*h*_*j*_) is true then *over-host-list* add *h*_*j*_
5: End for
6: For each *h*_*j*_ in *over-host-list*
7: While GMMHOD (*h*_*j*_) is true do
8: GMMMT (*h*_*j*_)
VM-migrate-list $\leftarrow vm$
9: End while
10: End for
11: For each *vm*_*i*_ in VM-migrate-list do
12: host = GMMOP (*vm*_*i*_*,host-list*)
13: Map add <*host, vm*_*i*_>
14: End for
15: Use the greedy strategy to shrink the host

## Experiment arrangement

We use CloudSim to simulate a cloud data center that contains 800 heterogeneous PMs and three instances of VMs. Tables 1 and 2 describe the resource configuration of VMs and PMs in the cloud data center respectively. The instance and configuration of PMs are from literature (https://www.spec.org/power_ssj2008/results/). In experiments, CloudSim creates VMs based on the user requests and randomly deploy them to PMs. Empirically, the two thresholds including ${T_{under}}$, ${T_{over}}$ can be set as ${T_{over}}$ = 0.8 and ${T_{under}}$ = 0.2. The learning parameter $a$ is set in terms of the experimental training results by the proposed algorithm 1 and AGM-VMC method. Here, by a large number of virtual machine consolidation experiments, it is found that when the learning parameter $a$ is set to 0.2, the experimental results are the best.

**Table 1:** Virtual machine instances.

Type	CPU (MIPS)	RAM (GB)
Micro instance	1,000	1
Medium instance	1,500	2
Large instance	2,800	4

**Table 2:** Physical machine instances.

Type	CPU	RAM (GB)
ProLiant DL380 G5	Intel Xeon L5430 2,666 MHZ	16
IBM system X3650	Intel Xeon X5570 2,933 MHZ	8

We use workload data from Alibaba Cluster Data V2018 (https://github.com/alibaba/clusterdata/blob/master/cluster-trace-v2018/trace_2018.md), a real dataset published by Alibaba Data Center in 2018, which records actual operational data and is referred to as the Alibaba dataset in this study. The Alibaba dataset contains a huge volume of historical records of both CPU and memory resources. Samples of task requests submitted on June 6, July 26, and August 15, 2018 are randomly selected here as the test workloads, with the workload characteristics detailed in Table 3.

**Table 3:** Alibaba data set workload characteristics.

No	Number of VMs	CPU		Memory	
		Mean (%)	St.dev (%)	Mean (%)	St.dev (%)
20180606-1	1,450	1.44	0.41	5.60	0.59
20180606-2	1,420	1.47	0.42	5.80	0.57
20180606-3	1,530	1.40	0.40	5.59	0.57
20180726-1	1,530	2.45	0.73	9.31	0.32
20180726-2	1,560	2.65	0.74	9.25	0.35
20180726-3	1,510	2.51	0.74	9.34	0.31
20180815-1	1,430	2.35	0.69	9.06	0.33
20180815-2	1,440	2.62	0.71	9.15	0.30
20180815-3	1,450	2.56	0.74	9.32	0.34

## Experiment results and analysis

We implement the complete VM consolidation algorithm AGM-VMC in Cloudsim, and compared with three VM consolidation methods including ND-RS, UP-BFD, and FFD-MMT. The ND-RS method uses a traditional Gaussian model (ND) for host overload detection and a random selection (RS) algorithm for VM selection. The UP-BFD method performs VM consolidation using the utilization prediction-aware best fit decreasing algorithm (Farahnakian, Pahikkala & Liljeberg, 2015). The FFD-MMT method uses the first-fit decreasing algorithm for VM placement and the minimum time migration policy for VM selection. For all of the compared VM consolidation methods, the pre-copy mechanism (Ibrahim, 2011) is employed to perform live VM migration.

### VM consolidation performance analysis

To compare the different VM consolidation manners, this section will analyze and evaluate the performance based on the proposed six evaluation indicators (Beloglazov & Buyya, 2012; Li, Yan & Yu, 2018). Table 4 shows their average of the four evaluation indicators of the four compared VM consolidation methods on Alibaba data set. As shown in Table 4, the SLAV, EC, ESV and VMM indicators of AGM-VMC method are the best one, and smaller than those of the other compared VM consolidation methods.

**Table 4:** The experimental results on the Alibaba cluster data.

	SLAV	EC	ESV	VMM	PDM	SLATAH
AGM-VMC	0.00007	94.18	0.00671	2,576	0.00893	0.80377
ND-RS	0.00101	107.31	0.08934	9,135	0.01951	2.37648
UP-BFD	0.00227	105.25	0.16911	6,060	0.04116	2.72919
FFD-MMT	0.00057	110.10	0.04557	15,292	0.01726	1.78532

The Service Level Agreement Violation (SLAV), also known as the violation rate of the service level agreement by a data center, reflects the data center’s QoS. Higher SLAV indicates a longer duration of service level agreement violation, namely a greater difficulty in guaranteeing QoS. Among the four methods above, the UP-BFD method has the highest SLAV, followed by the ND-RS method, the FFD-MMT method, and the AGM-VMC method in decreasing order. This is because the ND-RS, UP-BFD, and FFD-MMT methods are unable to predict workload trends when performing VM consolidation and thereby are more likely to cause host overloading and thus QoS degradation.

EC represents the energy consumption of each algorithm. As shown in Table 4, the EC of the AGM-VMC method is 10.05% to 14.50% lower than that of the other three methods, which indicates that the AGM-VMC method effectively reduces the data center energy consumption. The Alibaba dataset is a multi-resource dataset, but the mutual constraints between multiple resources are not imposed in the ND-RS, UP-BFD, and FFD-MMT methods. Consequently, the three methods fail to make full use of the resources in the PMs, which causes it to activate more PMs and thereby causes an increase in EC.

ESV, which is the product of EC and SLAV, is a comprehensive evaluation index of data center energy consumption and QoS, with smaller ESV representing better comprehensive performance of the data center in both aspects. The AGM-VMC method cause less SLAV and EC than the other three methods and thereby lower ESV. The other three methods are mutually compared as follows: (1) the FFD-MMT method causes higher EC but much lower SLAV than the other two methods, thereby leading to the lowest ESV of the three methods; (2) the UP-BFD method causes approximately the same EC as the ND-RS method but 2.25 times higher SLAV, thereby leading to higher ESV than the ND-RS method.

VMM represents the number of VM migrations. Since a migration VM will suspend services, reducing VMM is an important measure to improve the QoS of a data center. As shown in Table 4, the AGM-VMC method requires much less VMM than the other three methods and thereby effectively reduces the impact of VM migration on QoS of data center. The AGM-VMC method uses the adaptive Gaussian model to estimate the probability that the data center is in a balanced state of multi-resource utilization, which balances the workload of various resources and effectively keeps PMs free from the occurrence or risk of frequent overload. Moreover, this method avoids unnecessary VM migrations.

**Figure 3:**
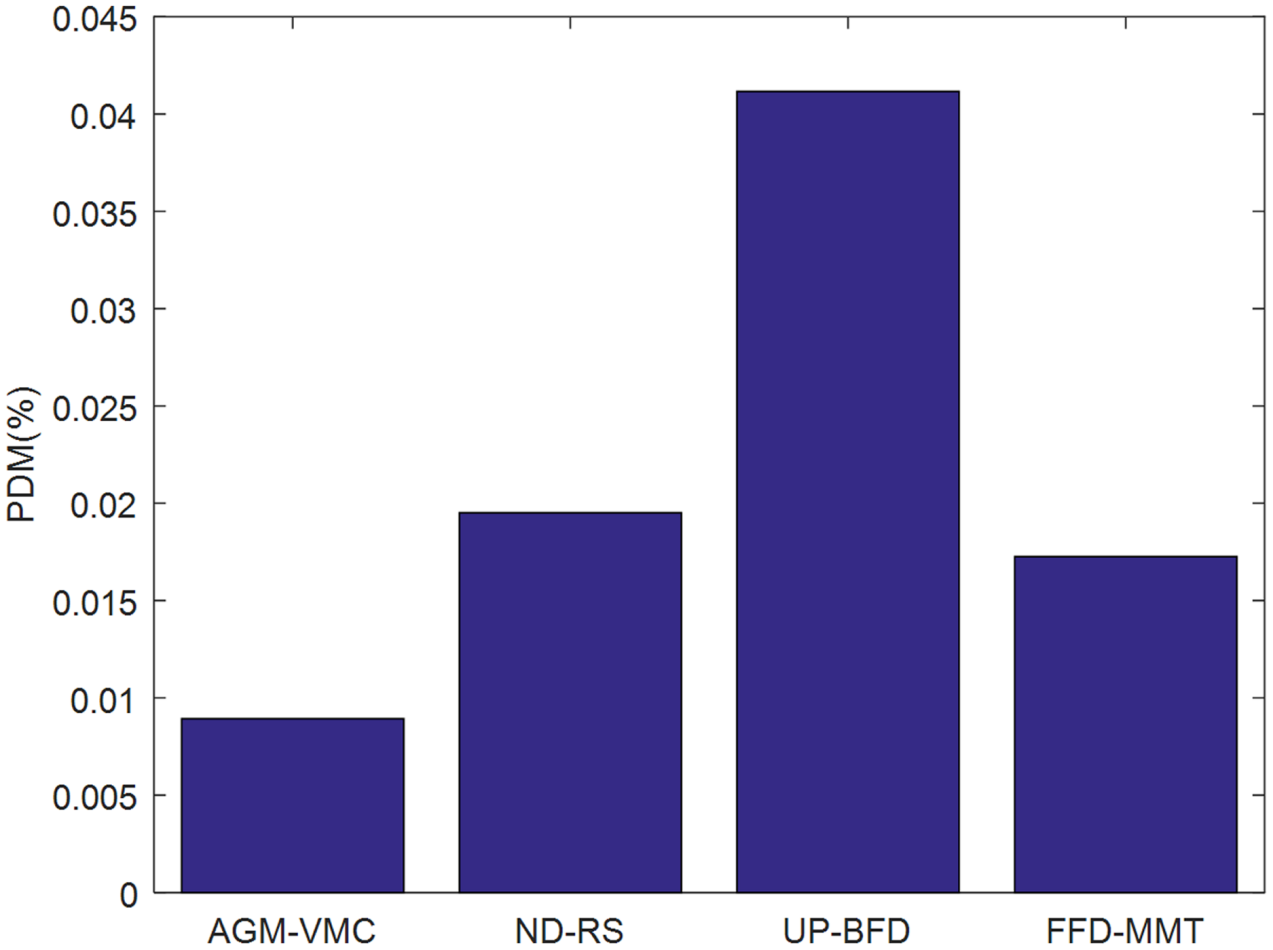
Comparison of PDM.

**Figure 4:**
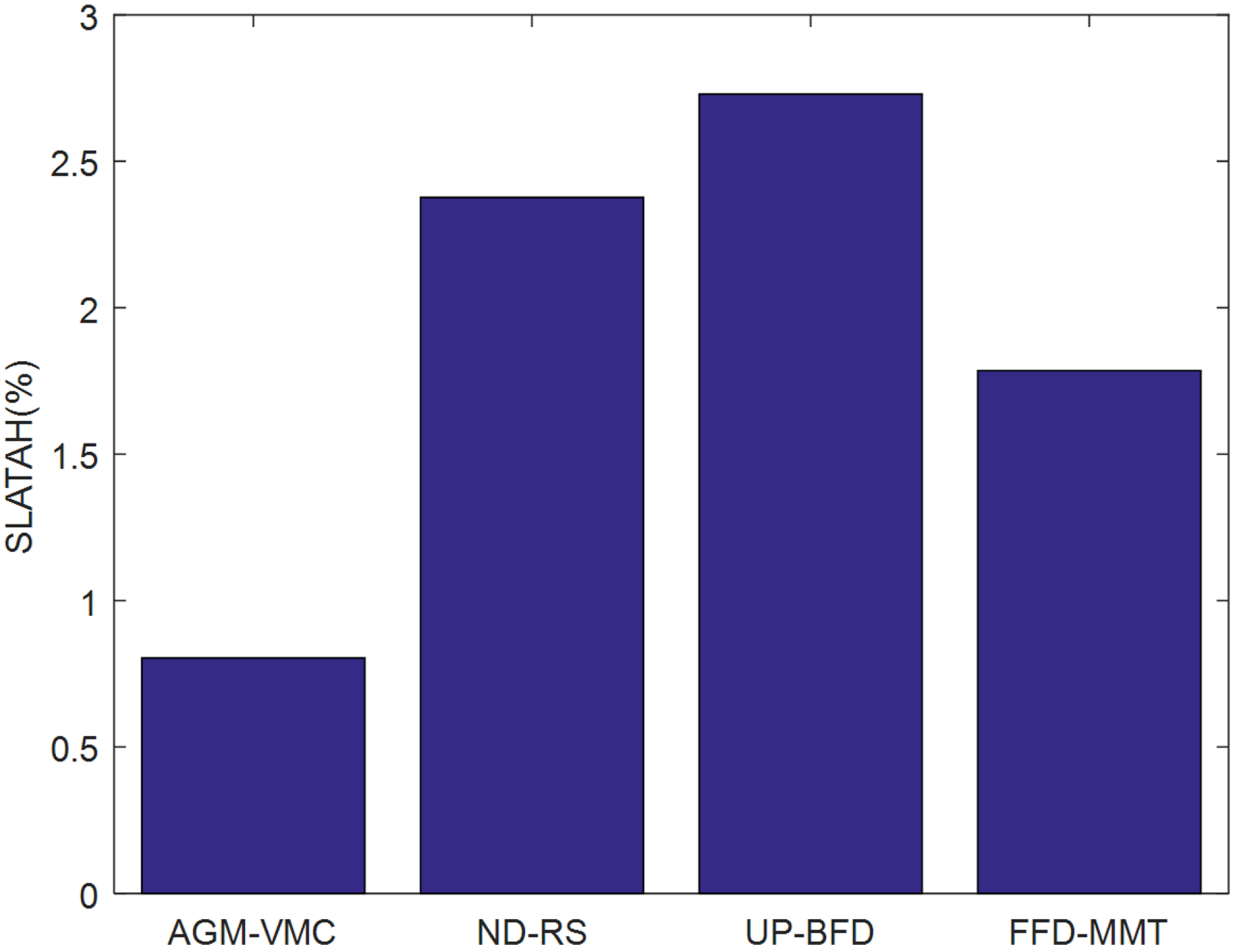
Comparison of SLATAH.

Figures 3 and 4 demonstrate the validation metrics PDM and SLATAH of the four methods, respectively, where PDM stands for performance degradation due to VM migrations, and SLATAH stands for service level agreement violation time per running host—a metric reflecting degradation of QoS due to the overloading risk of running hosts.

PDM is affected by both the number and duration of VM migrations during VM consolidation. In general, the lower the number of VM migrations and the shorter the VM migration time, the lower the PDM, *i.e*., the smaller the impact of VM migration on data center QoS. The AGM-VMC method comprehensively considers the factors that would minimize migration time and the factors that would maximize the probability of physical machines being in the ${N_{cpu}}{N_{ram}}$ state when selecting migration VMs. It aims to maximize the probability of physical machines being in a state of balanced resource utilization, prevent physical machines from overloading, ultimately lead to fewer VM migrations. Therefore, PDM is lower for the AGM-VMC method than for the other three methods, as shown in Fig. 3.

The longer duration that the resource requests of VMs cannot be fulfilled, the higher the SLATAH, and the greater the negative impact on QoS. The AGM-VMC method employs a strategy of maximizing the probability of PMs being in the ${N_{cpu}}{N_{ram}}$ state of resource utilization during VM placement, which indirectly helps balancing the workload of the data center, thereby effectively avoiding a situation in which running PMs fail to fulfill the resource utilization requests of VMs. In contrast, ND-RS just performs a random selection and placement strategy for VM consolidation, and the essential idea of ND-RS is relatively simple. UP-BFD examines VM consolidation by the scheme of utilization prediction-aware best fit decreasing. The idea of resource utilization prediction is advanced but difficult. As to the FFD-MMT, FFD algorithm for VM placement, which easily results in insufficient reserved resources in PMs, and suffers from reduction of QoS. MMT for VMs selection results in more VM migrations during their VM consolidation. Obviously, the ND-RS, UP-BFD, and FFD-MMT methods lack an ability to balance resource utilization and achieve balanced workload, so they result in larger SLATAH values than the AGM-VMC method.

### Resource utilization analysis

The four VM consolidation methods are compared in terms of changes in the number of running PMs, changes in the number of VM migrations, and changes in the utilization of physical machine resources as follows.

**Figure 5:**
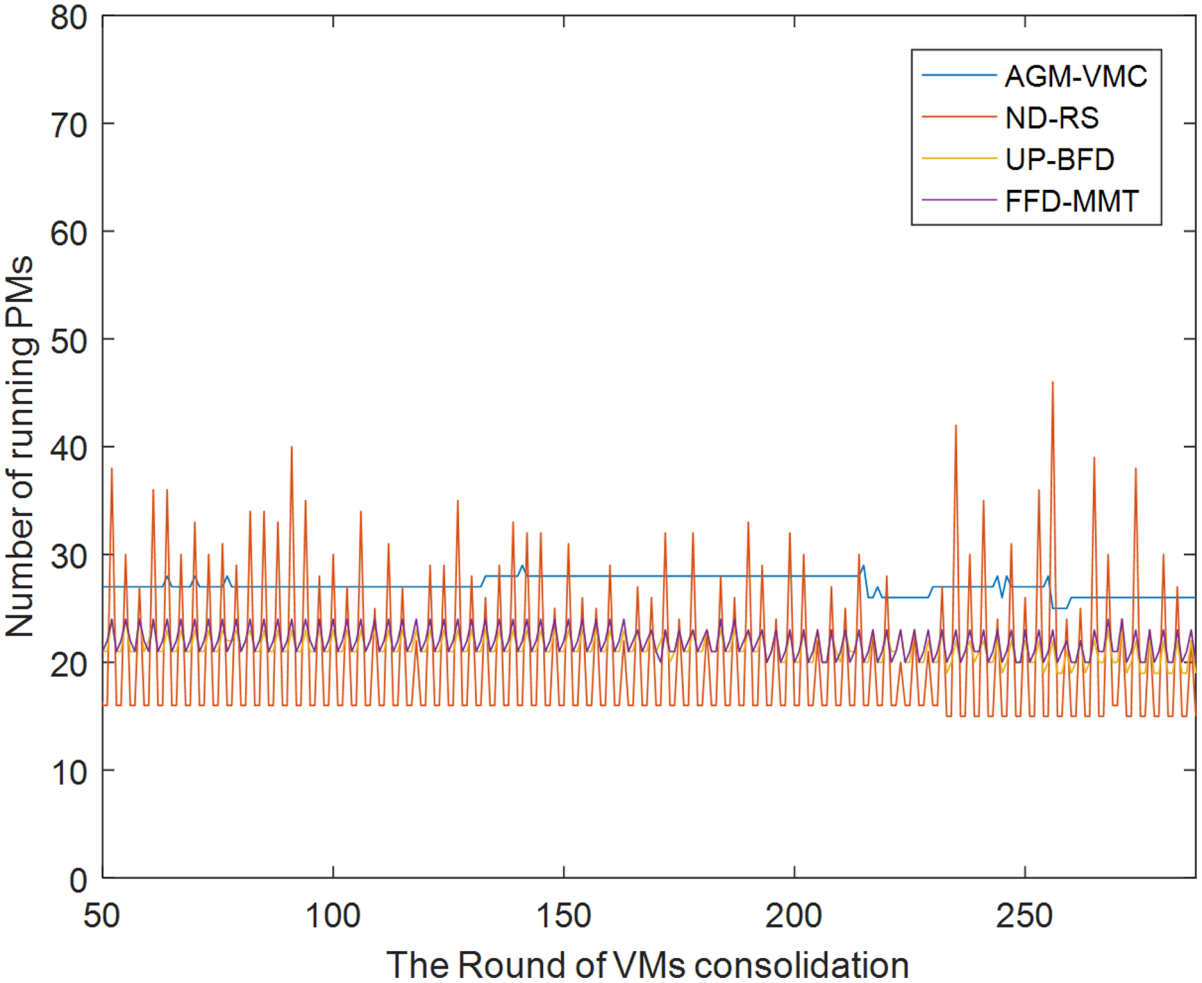
The changes in the number of running PMs.

**Figure 6:**
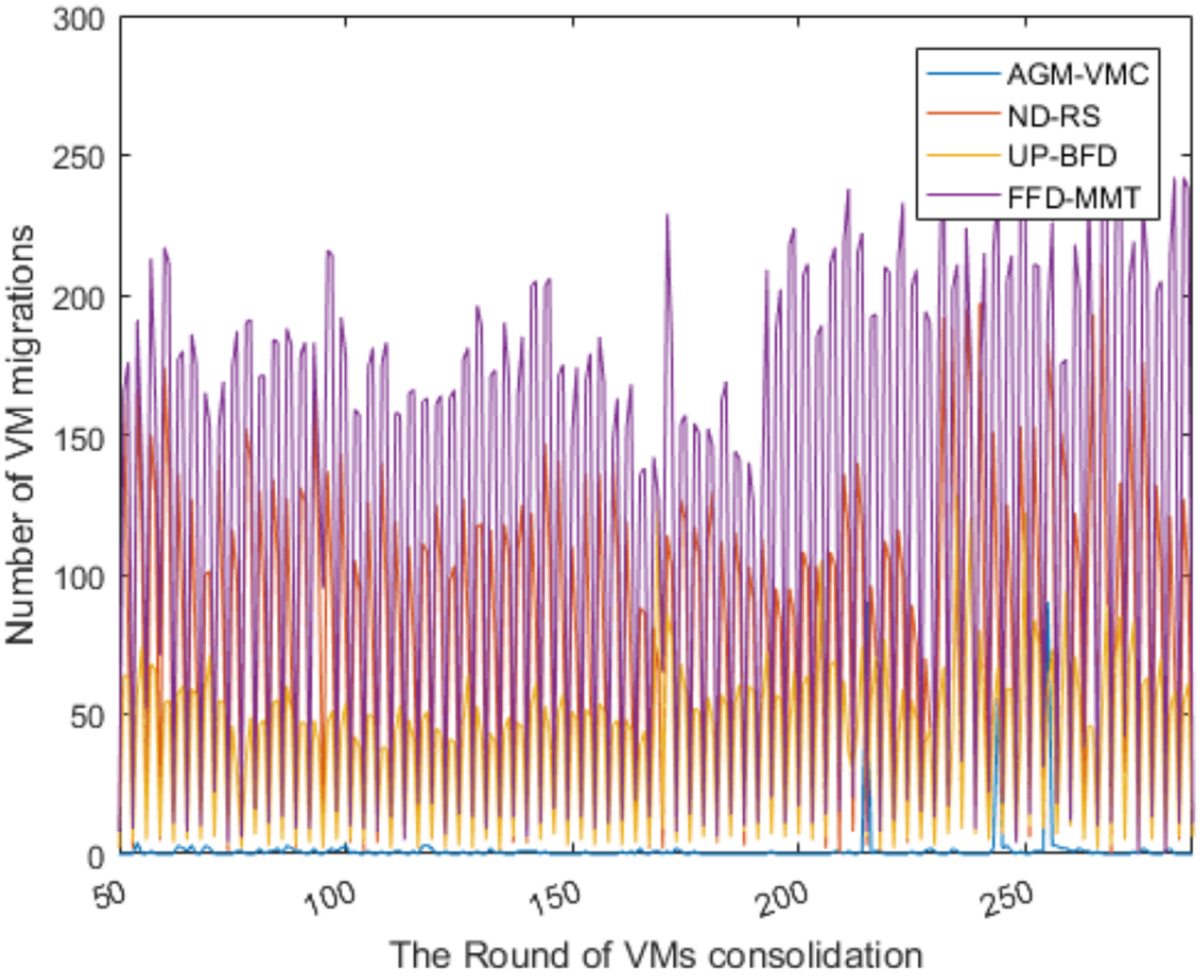
The changes in the number of VM migrations.

Figures 5 and 6 respectively show the number of running PMs and the number of VM migrations in the data center from the 50th VM consolidation to the 287th VM consolidation using each of the four methods. As shown in Fig. 5, the number of running PMs is slightly higher for the AGM-VMC method than for the other three methods. However, Fig. 6 shows that the number of VM migrations is far less for the AGM-VMC method than for the other three methods. These findings are mainly due to the fact that the AGM-VMC method is not only intended to minimize the number of running PMs during VM consolidation but also to balance resource utilization in running PMs to improve the comprehensive performance metrics of a data center such as load balance and QoS. In contrast, the ND-RS, UP-BFD, and FFD-MMT methods excessively reduce the number of running PMs while trying to avoid the negative impact of host overload on QoS. Therefore, they have to re-select destination hosts for a large number of migration VMs to be relocated. Excessive VM migrations do not only degrade QoS but also consume additional bandwidth resources. The AGM-VMC method, which attempts to achieve load balance in a data center, employs a strategy of slightly increasing the number of running PMs to balance the utilization of multiple resources, which reduces the number of VM migrations and thereby guarantee QoS.

**Figure 7:**
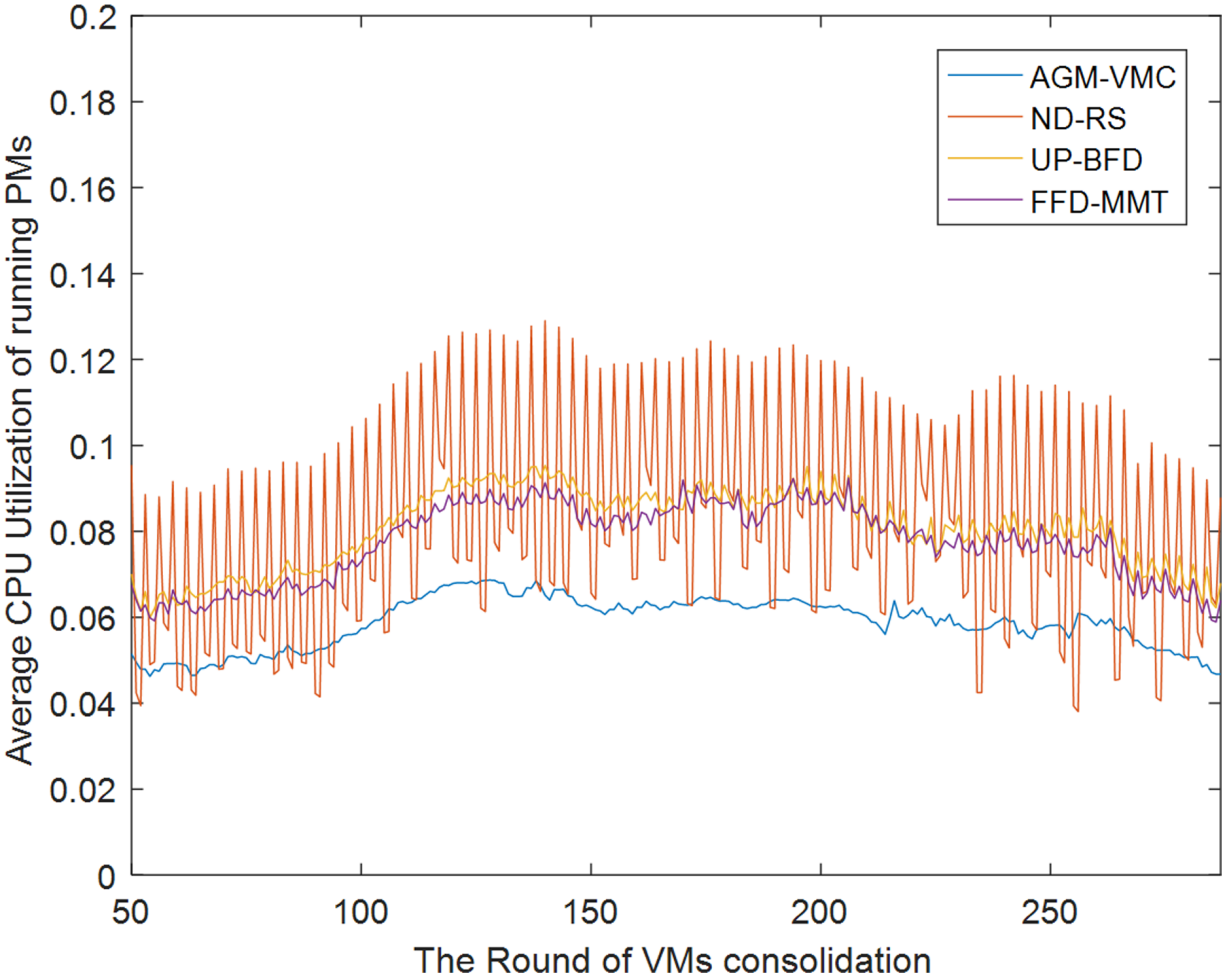
CPU utilization of running PMs.

**Figure 8:**
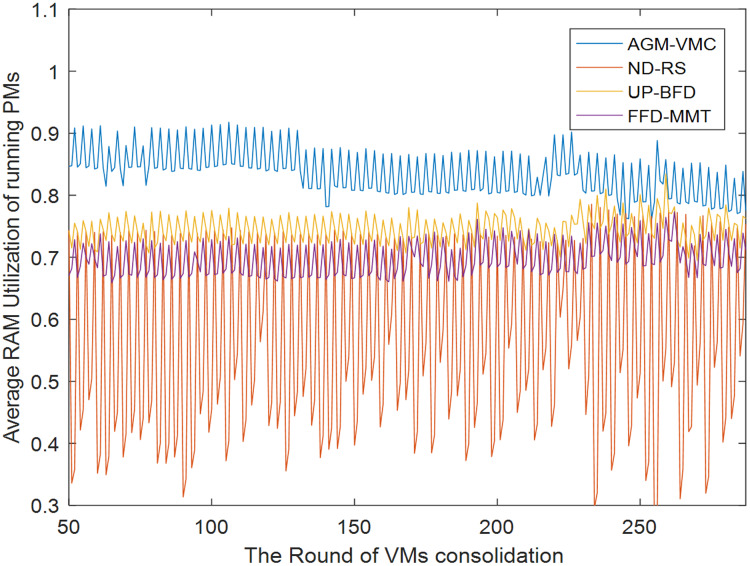
MEMORY utilization of running PMs.

Figures 7 and 8 demonstrate the CPU and memory resource utilization of the data center after VM consolidation with the GAM-VMC, ND-RS, UP-BFD, and FFD-MMT methods, respectively. As shown in Fig. 7, CPU resource utilization during VM consolidation is essentially in the same range for the ND-RS, UP-BFD, and FFD-MMT methods, smaller for the AGM-VMC method, and below 0.1 for all the four methods. This is attributed to the small volume of requests that are made by the Alibaba dataset for CPU resource utilization and consequently the small impact of VM consolidation on CPU resource utilization. Figure 8 demonstrates the variation of memory resource utilization in the data center for all the four consolidation methods. The memory resource utilization after VM consolidation is in the range 0.8–0.9 for the AGM-VMC method, which is higher than for the other three methods, especially the ND-RS method that achieves the lowest memory resource utilization. Figures 7 and 8 reveal that although the AGM-VMC method leads to slightly lower CPU utilization than the other three methods, it achieves more significant improvement of memory utilization. This demonstrates that AGM-VMC can dynamically migrate VMs according to the workload of hosts, and can improve the overall resource utilization of running PMs.

In summary, the AGM-VMC method effectively improves the energy consumption and QoS of a data center and reduces the number of VM migrations by balancing the utilization of various resources in PMs. As revealed by further analysis of host changes and VM changes during VM consolidation, the AGM-VMC method promotes load balancing among the PMs and reduces unnecessary VM migrations, thereby improving resource utilization and reducing resource wastage; this further helps reducing the energy consumption of a data center and in guaranteeing QoS.

## Conclusions

This paper proposes a coordinated optimization control mechanism for multi-resource utilization of PMs, and the AGM-VMP algorithm and AGM-VMC method based on this mechanism. The experiment results demonstrate their effectiveness in reducing the total number of VM migrations, decreasing energy consumption and maintaining the load balance of the cloud data center.

However, there are a few limitations that need to be further addressed in future works. First, the nine combination states of resource utilization only consider two kinds of resources. In fact, the constrained resources in the process of VM consolidation have others such as bandwidth, *etc.*; second, since cloud data center is a stochastic environment, it is hard to define the equilibrium state of resource utilization; then, VM consolidation can be triggered periodically by the cloud service provider, or triggered by the service monitor during the light workload time. Here, the cost of VM consolidation frequency is very challenging and need further study; finally, the three states of the two resource utilization and their parameters are currently determined by experience, and it is worth to develop a method that learn and define them dynamically and adaptively.

## Supplemental Information

10.7717/peerj-cs.852/supp-1 Supplemental Information 1 Code.

## Additional Information and Declarations

### Data Availability

The following information was supplied regarding data availability: The code is available in the Supplemental File.
